# miR-329b-5p Affects Sheep Intestinal Epithelial Cells against *Escherichia coli* F17 Infection

**DOI:** 10.3390/vetsci11050206

**Published:** 2024-05-08

**Authors:** Yeling Xu, Weihao Chen, Huiguo Yang, Zhenghai Song, Yeqing Wang, Rui Su, Joram M. Mwacharo, Xiaoyang Lv, Wei Sun

**Affiliations:** 1 College of Animal Science and Technology, Yangzhou University, Yangzhou 225009, China; yelingxu2001@163.com (Y.X.); 18552133709@163.com (W.C.); 2 Institute of Animal Husbandry, Xinjiang Academy of Animal Sciences, Urumqi 830013, China; yhg760924@163.com; 3 Dongshan Animal Epidemic Prevention Station, Wuzhong District, Suzhou 215100, China; 13451631997@163.com; 4 Suzhou Taihu Dongshang Sheep Industry Development Co., Ltd., Suzhou 215000, China; 15862308503@163.com; 5 Suzhou Stud Farm Co., Ltd., Suzhou 215200, China; surui99114074@163.com; 6 International Centre for Agricultural Research in the Dry Areas, Addis Ababa 999047, Ethiopia; j.mwacharo@cgiar.org; 7 Joint International Research Laboratory of Agriculture and Agri-Product Safety of Ministry of Education, Yangzhou University, Yangzhou 225009, China; dx120170085@yzu.edu.cn; 8 International Joint Research Laboratory in Universities of Jiangsu Province of China for Domestic Animal Germplasm Resources and Genetic Improvement, Yangzhou 225009, China

**Keywords:** *Escherichia coli* F17, micro RNA, sheep diarrhea, intestinal epithelial cells

## Abstract

**Simple Summary:**

Sheep diarrhea is one of the most common illnesses in sheep farming and diarrhea in sheep caused by *E. coli* occupies a significant proportion of them. *E. coli* F17 is one of the main members of *E. coli* causing diarrhea in sheep. This study is devoted to improving the resistance of sheep itself to *E. coli* F17. This study provides theoretical support for the solution of *E. coli* F17-caused diarrhea in sheep.

**Abstract:**

Diarrhea is the most common issue in sheep farms, typically due to pathogenic *Escherichia coli* (*E. coli*) infections, such as *E. coli* F17. microRNA, a primary type of non-coding RNA, has been shown to be involved in diarrhea caused by pathogenic *E. coli*. To elucidate the profound mechanisms of miRNA in *E. coli* F17 infections, methods such as *E. coli* F17 adhesion assay, colony counting assay, relative quantification of bacterial *E. coli* fimbriae gene expression, indirect immune fluorescence (IF), Cell Counting Kit-8 (CCK-8), 5-ethynyl-2′-deoxyuridine (EdU), Western blotting (WB), and scratch assay were conducted to investigate the effect of miR-329b-5p overexpression/knock-down on *E. coli* F17 susceptibility of sheep intestinal epithelial cells (IECs). The findings indicated that miR-329b-5p enhances the *E. coli* F17 resistance of sheep IECs to *E.coli* F17 by promoting adhesion between *E. coli* F17 and IEC, as well as IEC proliferation and migration. In summary, miR-329b-5p plays a crucial role in the defense of sheep IECs against *E. coli* F17 infection, providing valuable insights into its mechanism of action.

## 1. Introduction

Diarrhea is a common problem in sheep farms, which causes huge economic loss [1]. Enterotoxigenic *Escherichia coli* (ETEC), as one of the major classes of pathogenic *E. coli*, is considered to be the most common pathogenic bacteria of *E. coli*-associated diarrhea [2]. Mechanismly, ETEC can produce lipopolysaccharide (LPS) and enterotoxin, which interact to cause watery diarrhea in livestock. *Escherichia coli* F17 (*E. coli* F17), a major subtype of ETEC, has been detected in the feces of diarrheal lambs and calves in many areas and proven to be an important cause of diarrheal deaths in calves and lambs, and is the major cause of *E. coli* causing diarrhea in newborn alpacas [3,4,5,6]. This shows the widespread of *E. coli* F17 across multiple regions and species, and its study is becoming increasingly urgent.

The intestinal epithelium, consisting of IECS and cells of the immune system located within the intestinal mucosa, separates the internal environment of the intestine from the external environment and is one of the keys to the intestinal barrier [7]. IECs can maintain gut barrier health by modulating the immune response. They can regulate intestinal mucosal immunity through the production of cytokines and respond to cytokines secreted by immune cells through cell surface receptors [8]. IECs have been studied among many species. *LGR5* and *BMI1* increase the proliferation of porcine intestinal epithelial cells by stimulating Wnt/β-catenin signaling, which in turn promotes intestinal renewal [9]. *STIM1* accelerates porcine epithelial cell recovery through the *TRPC1* signaling pathway [10]. *STC-1* overexpression increases the antioxidant capacity of bovine IECs [11]. However, there are few studies on sheep IECs.

Small RNAs of about 20nt in length, known as microRNAs, are vital to numerous organisms. microRNAs have been proven to affect diverse cellular progress, including cell proliferation, apoptosis, migration, etc. [12,13,14]. miR-329, located at 14q32.31, has also been proved to affect diverse cellular progress, including cell proliferation, apoptosis, and migration [15,16,17,18]. Upregulation of miR-329-3p inhibited the proliferation of osteosarcoma cells and hepatocellular carcinoma cells [19,20]. Overexpression of miR-329-5P decreases fibroblast activation protein (*FAP*) expression, whereas circNOX4 adsorbs miR-329-5p to upregulate *FAP* and induce fibroblast activation [21]. miR-329b-5p, a member of the miR-329 family, is an miRNA that is differentially expressed in the sensitive and antagonistic groups based on pre-sequencing and has rarely been studied. miRNAs can be used as markers for inflammatory bowel disease (IBD); e.g., miR-16, 31, and 223 are all significantly different in IBD-related tests [22]. miRNAs also have the ability to promote epithelial regeneration after injury; for example, miR-31 promotes epithelial regeneration after injury by reducing inflammatory signaling [23]. A stable internal intestinal environment is essential for intestinal health. Down-regulation of MiR-30 inhibits intestinal epithelial cells and promotes intestinal epithelial cell differentiation [24]. miR-200b affects cell cycle progression by regulating *CCND1*, thereby promoting IECS proliferation [25]. miR-29b can affect IECs proliferation and influence intestinal homeostasis [26]. In addition to its vital role in cell growth and development and maintenance of intestinal health, microRNA has an important regulatory role in viral and bacterial diseases [27,28,29]. Although *E. coli* can colonize the brain and cause neuroinflammation, the inflammatory response induced by *E. coli* can be consistent through miR-155 and miR-146a [30]. Knocking down of miR-192 enhanced the expression of target genes and improved the adhesion of *E. coli* strains F18ab, F18ac, and K88ac, according to Sun et al. [31]. MiR-215 has been found by Dai et al. to target the *EREG*, *NIPAL1*, and *PTPRU* genes to modulate resistance to *E. coli* F18 in weaned piglets [32]. Ge et al. found that sheep β-Defensin 2 regulates *E. coli* F17 resistance in sheep intestinal epithelial cells through NF-κB and MAPK signaling pathways [33]. In contrast, little research has been carried out on microRNA regulation of *E. coli* F17 resistance in sheep.

To investigate the impact of miR-329b-5p on the resistance of sheep IECs to *E. coli* F17 infection, we transfected miR-329b-5p mimics and inhibitor and the corresponding controls into sheep IECs, respectively, and then carried out the *E. coli* F17 adhesion assay, colony counting assay, relative quantification of bacterial *E. coli* fimbriae gene expression, indirect IF, CCK8, EDU, WB and scratch assay to investigate the impact of miR-329b-5p on IECs. This article studies the resistance of sheep to *E. coli* F17 from the sheep themselves and provides a theoretical basis for solving the diarrhea caused by *E. coli* F17 in sheep.

## 2. Materials and Methods

### 2.1. Cell culture and Transfection

Sheep IECs used in this study were derived from previously established cell lines in our laboratory [33]. DMEM/F12 (HyClone, Logan, UT, USA) complemented with 15% fetal bovine serum (FBS) (Gibco, Grand Island, NY, USA) were used to culture IECs at 37 °C in 5% CO_2_, and 1% of penicillin–streptomycin was used to prevent the pollution. miR-329b-5p mimics, miR-329b-5p inhibitor, and mimics/inhibitor negative control (NC) and were designed and produced by GenePharma (Suzhou, China), mimics/inhibitor/NC were transfected into IECs at the cell density of 60~70% for 48 h before the subsequent experiment.

### 2.2. E. coli F17 Adherence Assay

*E. coli* F17 strain (DN1401) was obtained from Prof. Dongfang Shi’ lab at the Northeast Agricultural University. *E. coli* F17 were inoculated to LB solid culture medium, inverted, and incubated overnight, then single colonies were picked and shaken at 37 °C for 4 h. Transfected cells were added to *E. coli* F17 and DMEM/F12 in a 1:1 configuration and incubated at 37 °C for 3 h. Then, the adhesion capacity of *E. coli* F17 to sheep IECs via colony counting assay, relative quantification of bacterial *E. coli* fimbriae gene expression, and indirect immune fluorescence were carried out.

### 2.3. Cell Proliferation

After cell transfection, the OD values of the cells at 0 h, 24 h, 48 h, and 72 h were detected using the Cell Counting Kit-8 (CCK-8, Vazyme, Nanjing, China). Cell proliferation was detected after 48 h using an EdU kit (RiboBio, Guangzhou, China) according to the instructions.

### 2.4. RT-qPCR

The transfection efficiency of miR-329b-5p mimics and miR-329b-5p inhibitor was tested using the stem-loop method and ChamQ SYBR qPCR Master Mix kit (Vazyme, Nanjing, China). IECs were infected with *E. coli* F17 48 h after transfection. DNA was extracted using TIANamp Bacteria DNA kit (TIANGEN, Beijing, China). Extracted DNA was used as a template and *GAPDH* as an internal reference to detect the expression of the fimbriae gene F17b-A and F17b-G using the *E. coli* F17 fimbriae gene primers (Table 1). To verify the effect of miR-329b-5p on tight junctions between IECs, the expression of mRNA levels of the *vimentin*, which are associated with tight junctions, was examined. The role of miR-329b-5p on IEC’s proliferation was further investigated using RT-qPCR detection of proliferation markers, including proliferating cell nuclear antigen (*PCNA*) and cyclin D1 (*CCND1*). *GAPDH* was used as a reference gene to estimate the relative expression level of the target gene.

### 2.5. Western Blot

Using RIPA lysate (Beyotime, Shanghai, China), cells were lysed, and proteins were collected. We used a BCA kit to detect the protein concentration and performed the protein denaturation according to protein concentration. After SDS-PAGE electrophoresis, proteins were transferred to PVDF membranes. PVDF membranes were incubated with primary antibodies with PCNA (1:2000), vimentin (1:10,000), *GAPDH* (1:5000), and then with rabbit secondary antibodies and mouse secondary antibodies. Detection was performed using the ECL Western Blot kit (BioSharp, Hefei, China).

### 2.6. Scratch Assay

Sheep IECs were inoculated in 12-well plates and transfected when the cells reached 60%. Forty-eight hours later, a line was traced across the 12-well plate, and 2% FBS medium was applied. Using a microscope, images were collected at 0 and 12 h. The migratory capacity of the cells was analyzed based on the healed area of the scratch.

### 2.7. Statistical Analysis

All experiments were performed using the −2^ΔΔCT^ method. Software for statistical analysis, SPSS 26.0, was utilized. An independent sample *t*-test was used to perform variance analysis and significance test. All experimental data were expressed as mean ± SEM * *p* < 0.05 and ** *p* < 0.01. A Benjamini–Hochberg correction was performed.

## 3. Results

### 3.1. Effects of miR-329b-5p Mimics and miR-329b-5p Inhibitor

**Figure 1:**
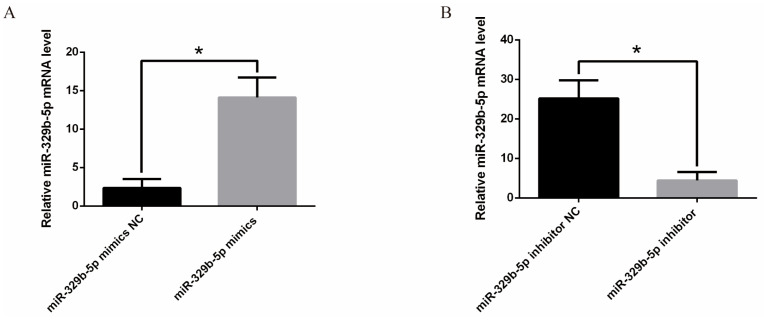
Efficiency verification of miR-329b-5p mimics and miR-329p-5p inhibitors. (**A**) Transfection of miR-329b-5p mimics and NC into sheep IECs. Expression of miR-329b-5p was detected by RT-qPCR 48 h after transfection. (**B**) Transfection of miR-329b-5p inhibitor and inhibitor NC into sheep IECs. Expression of miR-329b-5p was detected by RT-qPCR 48 h after transfection. * *p* < 0.05.

The mRNA expression level of miR-329b-5p in IECs was detected by RT-qPCR. The expression of miR-329b-5p mimics was significantly increased at the mRNA level after transfection with miR-329b-5p mimics (*p* < 0.05) (Figure 1A), whereas the opposite was true after transfection with miR-329b-5p inhibitor (*p* < 0.05) (Figure 1B). The above results indicate that the miR-329b-5p mimics and miR-329b-5p inhibitors are efficiently transfected and can be used for subsequent assays.

### 3.2. miR-329b-5p Influences the E. coli F17 Susceptibility of Sheep IEC s

*E. coli* F17 infection assay was performed in sheep IECs transfected with miR-329b-5p mimics, miR-329b-5p NC, and miR-329b-5p inhibitor, miR-329b-5p inhibitor NC, and the effects of miR-329b-5p on *E. coli* F17 infection were verified by *E. coli* F17 colony counting and fimbriae gene RT-qPCR respectively and immunofluorescence assay to verify the effect of miR-329b-5p on sheep IECs against *E. coli* F17 infection.

**Figure 2:**
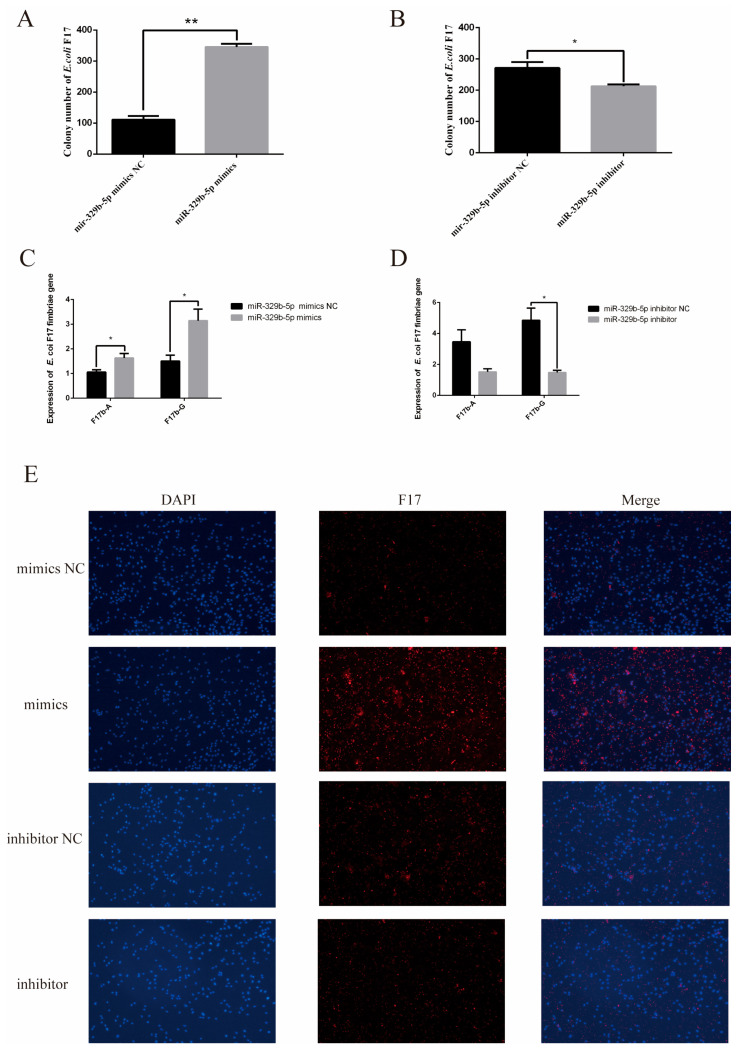
miR-329b-5p affects susceptibility of sheep IEC to *E. coli* F17. (**A**) Colony count of *E. coli* F17 after up-regulation of miR-329b-5p. (**B**) Colony count of *E. coli* F17 after down-regulation of miR-329b-5p. (**C**) RT-qPCR of *E. coli* F17 fimbriae gene after up-regulation of miR-329b-5p. (**D**) RT-qPCR of *E. coli* F17 fimbriae gene after down-regulation of miR-329b-5p. (**E**) In the immunofluorescence assay, cells were observed under the fluorescence microscope (100×). * *p* < 0.05 and ** *p* < 0.01.

The results of colony counting showed (Figure 2A,B) that the *E. coli* F17 adhering to the IECs in the miR-329b-5p mimics transfection group was significantly higher than that in the miR-329b-5p mimics NC (*p* < 0.01), and correspondingly, the *E. coli* F17 adhering to the IECs in the miR-329b-5p inhibitor transfection group was significantly lower than that of miR-329b-5p inhibitor NC (*p* < 0.05).

The RT-qPCR of the fimbriae gene was performed targeting F17b-A and F17b-G, respectively, and the results showed (Figure 2C) that both F17b-A and F17b-G fimbriae genes were significantly higher in the miR-329b-5p mimics transfection group than in the miR-329b-5p mimics NC (*p* < 0.05). In contrast (Figure 2D), the miR-329b-5p inhibitor transfected group F17b-A fimbriae gene was lower than the miR-329b-5p inhibitor NC, whereas F17b-G fimbriae gene was significantly lower than the miR-329b-5p inhibitor NC (*p* < 0.05).

Immunofluorescence results showed (Figure 2E) that more *E. coli* F17 than miR-329b-5p mimics NC adhered to the miR-329b-5p mimics transfection group, whereas less *E. coli* F17 than miR-329b-5p inhibitor NC adhered to the miR-329b-5p inhibitor transfection group. In summary, miR-329b-5p has an important effect on the ability of IECs to resist *E. coli* F17. In addition, we detected the expression of *vimentin* in IECs at the mRNA level after transfection with miR-329b-5p mimics and NC as well as miR-329b-5p inhibitor and inhibitor NC by RT-qPCR assay, and we detected the expression of the *vimentin* gene at the protein level by WB.

**Figure 3:**
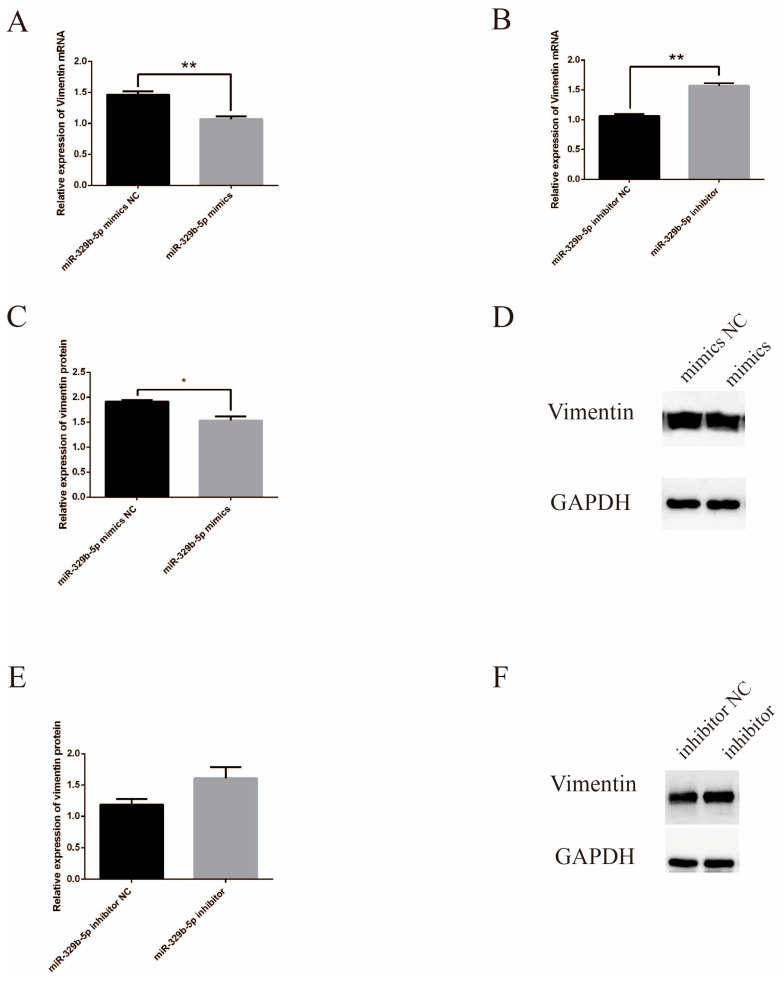
Effect of miR-329b-5p on *vimentin* expression. (**A**) RT-qPCR assay of *vimentin* expression after up-regulation of miR-329b-5p. (**B**) RT-qPCR assay of *vimentin* expression after down-regulation of miR-329b-5p. (**C**,**D**) WB of *vimentin* expression after up-regulation of miR-329b-5p. (**E**,**F**) WB of *vimentin* expression after down-regulation of miR-329b-5p. * *p* < 0.05 and ** *p* < 0.01.

Figure 3A showed that after overexpression of miR-329b-5p, *vimentin* was extremely significantly lower than the NC. After inhibition of miR-329b-5p, *vimentin* was significantly higher than the inhibitor NC at the mRNA level (Figure 3B). *Vimentin* was significantly lower at the protein level after transfection with miR-329b-5p mimics than NC, while after transfection with miR-329b-5p inhibitor, the protein levels of *vimentin* were higher than inhibitor NC (Figure 3C–F) (A detailed picture of the WB was shown in Figure S1). In. In summary, up-regulation or down-regulation of miR-329b-5p affects *vimentin* expression at both the mRNA and protein levels.

### 3.3. miR-329b-5p Suppress the Proliferation of IECs

**Figure 4:**
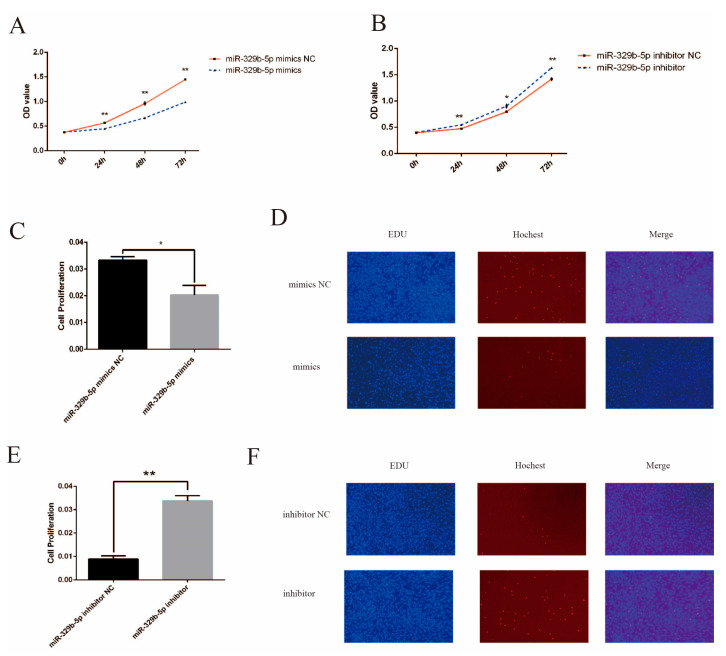
Effect of miR-329b-5p on proliferation of IECs. (**A**) CCK-8 assay after up-regulation of miR-329b-5p. (**B**) CCK-8 assay after down-regulation of miR-329b-5p. (**C**,**D**) EDU assay after up-regulation of miR-329b-5p (100×). (**E**,**F**) EDU assay after -regulation of miR-329b-5p (100×). * *p* < 0.05 and ** *p* < 0.01.

In order to investigate the effect of miR-329b-5p on the proliferation of sheep IECs, we performed CCK-8 and EDU assays. The results of CCK-8 assay showed (Figure 4A,B) that the OD values of cell growth after transfection with miR-329b-5p mimics were extremely significantly lower than that of the corresponding control group at 24/48/72 h (*p* < 0.01), while OD values of cell growth at 24/72 h after transfection with miR-329b-5p inhibitor were highly significantly higher than the corresponding control group (*p* < 0.01), and significantly lower at 48 h (*p* < 0.05). The results of the EDU assay showed (Figure 4C–F) that the rate of EDU positive cells detected after transfection by miR-329b-5p mimics was significantly lower than the corresponding control group (*p* < 0.05), and the rate of EDU-positive cells detected after transfection using miR-329b-5p inhibitor was significantly higher than the corresponding control group (*p* < 0.01).

**Figure 5:**
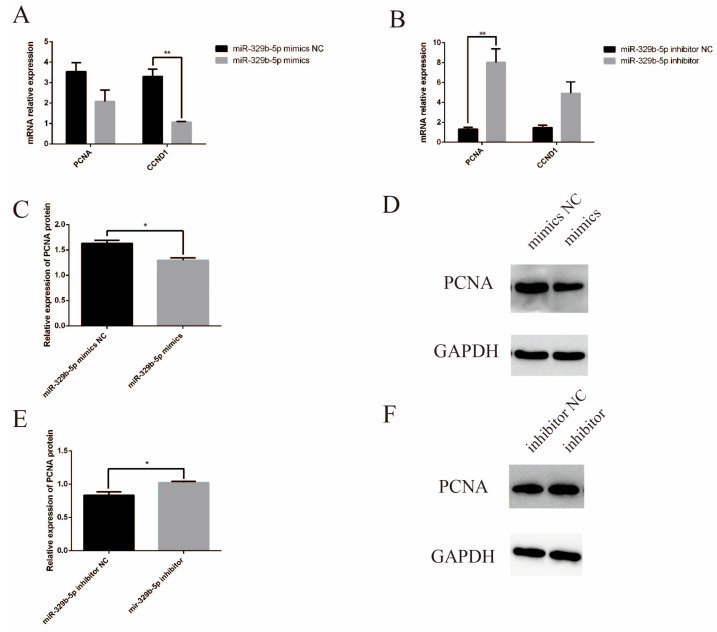
Effect of miR-329b-5p on proliferation of IECs detected by proliferation markers. (**A**) mRNA relative expression of *PCNA* and *CCND1* after up-regulation of miR-329b-5p. (**B**) mRNA relative expression of *PCNA* and *CCND1* after down-regulation of miR-329b-5p. (**C**,**D**) Protein expression of *PCNA* after up-regulation of miR-329b-5p. (**E**,**F**) Protein expression of *PCNA* after down-regulation of miR-329b-5p. * *p* < 0.05 and ** *p* < 0.01.

In addition, the expression of *PCNA* and *CCND1* at the mRNA level was reduced after miR-329p-5p mimic transfection compared to the miR-329b-5p mimic NC group (Figure 5A), while the opposite was true for miR-329b-5p inhibitor (Figure 5B). Western blot results showed (Figure 5C–F) that the expression of PCNA protein, a marker associated with cell proliferation, was significantly decreased (*p* < 0.05) after miR-329b-5p mimics transfection, while *PCNA* expression was significantly increased (*p* < 0.05) after miR-329b-5p inhibitor transfection (A detailed picture of the WB was shown in Figure S2). In summary, up-regulation of miR-329b-5p inhibits sheep IEC’s proliferation, while down-regulation of miR-329 promotes sheep IEC’s proliferation.

### 3.4. miR-329b-5p Suppress the Migration of IECs

**Figure 6:**
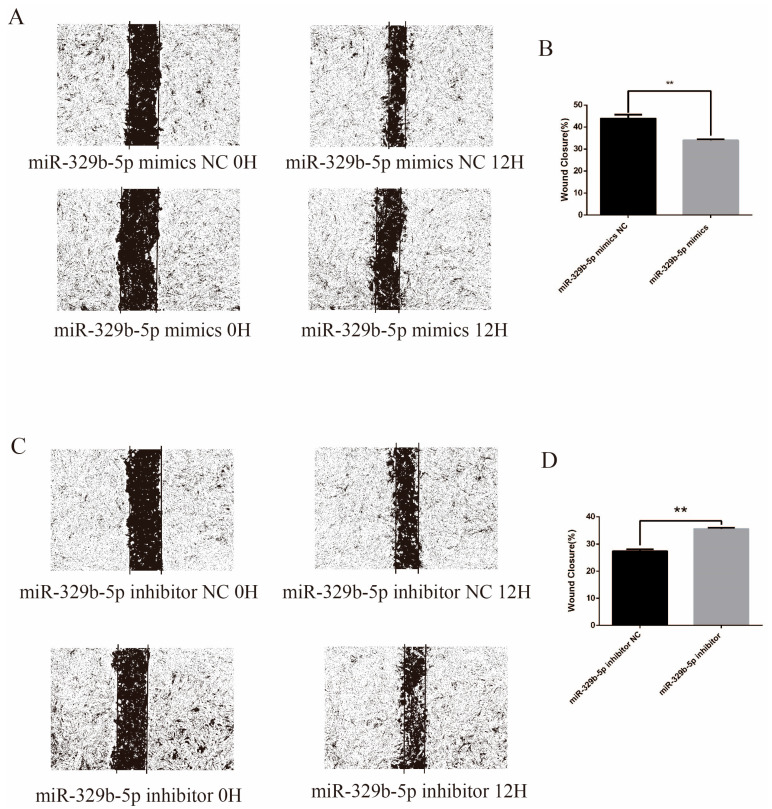
Effect of miR-329b-5p on migration of IECs. (**A**,**B**) Scratch assay after up-regulation of miR-329b-5p. (**C**,**D**) Scratch assay after down-regulation of miR-329b-5p. ** *p* < 0.01.

In this study, the effect of miR-329b-5p on the migration of sheep IECs was examined by cell scratch assay. Figure 6 showed that the wound healing speed of cells in the transfected miR-329b-5p mimics group was significantly slower than that of the miR-329b-5p mimics NC (*p* < 0.05), whereas the wound healing speed of cells in the transfected miR-329b-5p inhibitor group was highly significantly faster than that of the miR-329b-5p inhibitor NC (*p* < 0.05), thus indicating that miR-329b-5p can inhibit the migration of sheep IECs.

## 4. Discussion

One of the most prevalent illnesses on sheep farms is *Escherichia coli*-caused sheep diarrhea. ETEC is one of the major pathogenic *E. coli*. ETEC mainly colonizes the proximal small intestine, and ETEC colonization of the small intestine is predominantly dependent on the expression of several different protein surface structures, which are commonly referred to as colonization factors (CFs) [34]. One of the primary members of the ETEC family is *E. coli* F17. *E. coli* F17 is widespread in Southern Peru, Russia, Iran, and other places around the world [3,6,35]. *E. coli* F17 fimbriae are mainly composed of the pilin F17A and F17G adhesin. F17G adhesin is one of the most important adhesion factors that cause diarrhea in ruminants by mediating on the intestinal microvilli [36]. The study by Ana Umpiérrez et al. tested calves for *Escherichia coli* adhesin-related genes with a high abundance of F17G [37]. *E. coli* causes host damage by colonizing mucosal sites, then evading host defenses and beginning to multiply. Although the intestinal flora itself competes for nutrients, *E. coli* has specific fimbrial antigens that enhance its intestinal colonization and adherence to the small intestinal mucosa, causing host damage [38]. Therefore, the present study focused on enhancing the resistance of sheep IECs to *E. coli* F17 adhesion through a host receptor cell perspective. This study shows that IECs were found to have enhanced resistance to F17 adhesion after inhibition of miR-329b-5p expression, both in the detection of the *E. coli* F17 fimbriae gene, the *E. coli* F17 colony counting assay, and indirect immunofluorescence.

miRNAs have been the subject of numerous studies in gut homeostasis, inflammation, and damage repair. Increasing evidence has shown that miRNAs play a vital role in intestinal innate immunity, e.g., mice with miR-223 deficient in macrophages and dendritic cells exhibit a strong pro-inflammatory phenotype [39]. Liang et al. showed that mice with knockout of miR-146b exhibited enhanced M1 macrophage polarization [40]. miRNAs can also be involved in adaptive immunity. miR-221 and miR-222, specifically lost in T cells, promote colitis infection in mice [41]. In addition, it has been suggested that miRNAs may affect gut microbiota composition and function, thereby influencing gut homeostasis and host health [42]. We therefore speculate that miR-329b-5p may influence the ability of sheep to resist *E. coli* F17 infection by affecting intestinal innate immunity, adaptive immunity, or gut microbes. Lipid metabolism and immune function in the bovine intestine may be altered by miRNAs, which may affect the interactions between the host and *E. coli* O157 most, leading to excessive shedding of *E. coli* O157 in cattle, affecting the farm environment and contaminating farm crops [43]. A. Jaeger et al. found that many miRNAs were significantly up-regulated in porcine mammary epithelial cells attacked for 3 and 24 h [44]. Chen et al. conducted RNA sequencing on the ileal tissues of sheep that were either *E. coli* F17-sensitive or antagonistic, identifying differentially expressed miRNAs and circRNAs, and constructing the corresponding ceRNA networks [45]. Therefore, in this study, we selected the screened differentially expressed miR-329b-5p to investigate the role of miRNAs against *E. coli* infection in sheep. MiR-329 has been found to inhibit the proliferation, invasion, and migration of melanoma cells, which is similar to its function in small intestinal epithelial cells. This suggests that similar mechanisms may also exist in small intestinal cells, such as genes such as *HMGB2*. According to reports, the *HMGB2* gene is associated with proliferation, apoptosis, and tumors. In melanoma cells, silencing of the *HMGB2* gene can weaken cell viability, and there is a targeted relationship between miR-329 and the *HMGB2* gene, which can negatively regulate the *HMGB2* gene and inhibit β- catenin pathway, thereby regulating cellular life activities [46].

Intestinal barriers include mechanical, immune, biological, and chemical barriers [47]. The mechanical barrier is one of the crucial components of the intestinal barrier and is maintained mainly by IECs and intercellular junctions [48]. Massive epithelial cell death leads to increased intestinal permeability and microflora dysbiosis, thus providing an opportunity for pathogens to breach the intestinal barrier [49]. In addition, small intestinal mucus contains high concentrations of antimicrobial peptides and proteins secreted by panniculus cells and enterocytes, which kill or trap bacteria, thus preventing the epithelial cells from coming into contact with the bacteria [50]. miRNAs regulate intestinal epithelial homeostasis by altering IEC’s proliferation, migration, and cell–cell interactions [51]. miR-138-5p overexpression inhibits pyroptosis, promotes tight junctions, and ameliorates intestinal barrier breakdown [52]. Inhibition of miR-379-5p promotes IEC’s proliferation, restores barrier function, and improves survival after intestinal injury [53]. This is consistent with the current study’s results, which found that inhibition of miR-329b-5p promotes the proliferation of IECs, which are important for the stability of the intestinal barrier, based on CCK-8, EDU. *PCNA* is a gene that is extremely important in organisms and is involved in DNA replication, damage repair, and transcription in organisms. In addition, *PCNA* is able to participate in various cellular activities and is involved in energy metabolism [54]. *PCNA* is a cofactor for DNA polymerase δ, which is involved in cell proliferation [55]. *PCNA* expression is elevated in the G1/S phase of cells and is low in quiescent and senescent cells [56]. Wang et al. found that *PCNA* may interact with *KCTD10* and have an effect on cell proliferation [57]. It has been suggested that growth factors or damaged DNA may induce an increase in *PCNA* [56]. *CCND1* specifically regulates the cell cycle and functions primarily in the nucleus. Both *CCND1* and *PCNA* are commonly used as markers to detect cell proliferation. *CCND1*, located on chromosome 11q13, is a member of the *CCND* family and is commonly used as a marker for cell proliferation and cancer. Its primary function is to facilitate the transition of cells from the G1 phase to the S phase, resulting in the acceleration of cell proliferation and playing a crucial role in cell migration [58,59]. It has been confirmed that *CCND1* can be regulated by miR-193a-3p to inhibit the rapid growth and metastasis of pancreatic cancer [60]. CircCCND1 can interact with *HuR* and miR-646, synergistically enhancing the stability of CCND1 mRNA, thereby promoting LSCC cell proliferation [61]. These results also demonstrate the regulatory ability of *CCND1* on cell proliferation and its reliability as a biomarker. *CCND1* can activate the MAPK/PI3K-AKT signaling pathway [62]. In this study, we examined the expression of proliferation markers *CCND1* and *PCNA* at the mRNA level and protein level and found that after down-regulation of miR-329b-5p, the expression of *PCNA* and *CCND1* increased, which was consistent with the results of CCK-8 and EDU assays. Thus, inhibition of miR-329b-5p promotes the proliferation of intestinal epithelial cells, which leads to a more stable intestinal barrier and increases the ability of intestinal epithelial cells to resist *E. coli* F17 infection.

*Vimentin* is an intermediate filament protein involved in a variety of functions in living organisms, including maintenance of cytoskeletal integrity, cytokinesis, intracellular signal transduction, cell adhesion, and cell migration [63,64,65,66,67]. *Vimentin* is also a typical marker of epithelial–mesenchymal transition (EMT) [68]. During EMT, epithelial cells differentiate functionally and behaviorally into the mesenchymal cell type, which is essential for tissue regeneration and wound healing [69]. Knocking mice out of *vimentin* results in mice with defects associated with the ability to repair wound damage [70]. It has been demonstrated that *vimentin* deficiency prevents normal wound healing, causing slow and incomplete tissue recovery [71]. Yang et al. showed that overexpression of CLDN6 in human triple-negative breast cancer cells inhibited cell proliferation and resulted in downregulation of the mesenchymal marker *vimentin* [72]. Overexpression of UNC13C in the human tongue squamous carcinoma cell downregulated *vimentin* expression [73]. In addition, miR-17-5p overexpression reduced *vimentin* expression at the mRNA level and protein level [74]. This is consistent with the current study’s results, which revealed that down-regulation of miR-329b-5p expression in IECs up-regulates *vimentin* expression at both the mRNA and protein level.

One of the foundations for establishing and maintaining the normal organization of an organism is cell migration [75]. Cell migration plays an emphatic role in tissue homeostasis, immune response, and wound repair. When damage occurs in the body, cells migrate quickly to repair the damage [76]. The results of Xu et al. found that miR-301a knockout mice had reduced macrophage migration capacity, which affected subsequent damage repair in damaged tissues [77]. Liu et al. discovered that miR-874-3p upregulation diminishes the migration capability of osteosarcoma cells [78]. This resembled the results of the present study, where the results of the scratch assay indicated that down-regulation of miR-329b-5p improves the migration of IEC, thereby enhancing the resistance of IEC to *E. coli* F17 in the infection.

Although, in this study, we found that up-regulation of miR-329b-5p decreased the ability of IECs to resist *E. coli* F17 by *E. coli* F17 fimbriae gene assay, colony counting, and immunofluorescence and inhibited the proliferation and migration of IECs. However, this study still has shortcomings, as it did not include any cellular attack during the testing of cell proliferation and migration ability. As the cells after tapping, with higher mortality rate, could not support proliferation and migration-related experiments, the experimental protocols could be subsequently optimized for further attempts to assay cell proliferation and migration ability after attacking. miRNAs often act on cells by targeting genes, while miRNAs can often be adsorbed by circRNAs or bind to lncRNAs to play corresponding roles. In contrast, the present study lacks relevant research, which can be followed up with the target genes of miR-329b-5p to further investigate the molecular mechanisms affecting the resistance of sheep to *E. coli* F17 infection. This study is currently conducted only at the cellular level and lacks animal in vivo tests, which can be followed up with in vivo knockout tests for validation.

## 5. Conclusions

The up-regulation of miR-329b-5p can improve the susceptibility of sheep IECs to *E. coli* F17, while the down-regulation of miR-329b-5p inhibits the proliferation and migration of sheep IECs. In conclusion, miR-329b-5p plays a crucial role in the resistance of sheep IECs against *E. coli* F17 infection. This study offers a scientific foundation for elucidating the mechanism of miR-329b-5p on the *E. coli* F17 susceptibility of sheep IECs.

## Figures and Tables

**Table 1:** Sequence information of genes used in RT-qPCR.

Gene Name	Sequences (5′→3′)	Product Length/bp	Accession No.
F17b-A	F: CAACTAACGGGATGTACAGTTTC R: CTGATAAGCGATGGTGTAATTAAC	323	L14318.1
F17b-G	F: CGTGGGAAATTATCTATCAACG R: TGTTGATATTCCGTTAACCGTAC	615	L14319.1
*Vimentin*	F: CTGCTAACCGCAACAACGAC R: TAGTCCCTTTGAGCGCATCC	108	XM_004014247.6
*PCNA*	F: TCTGCAAGTGGAGAACTTGGAA R: AGGAGACAGTGGAGTGGCTT	162	XM_004014340.5
*CCND1*	F: CCGAGGAGAACAAGCAGATC R: GAGGGTGGGTTGGAAATG	91	XM_027959928.2
*GAPDH*	F: TCTCAAGGGCATTCTAGGCTAC R: GCCGAATTCATTGTCGTACCAG	151	XM_060411593.1

## Data Availability

All data are contained within the article.

## Supplementary Materials

The following supporting information can be downloaded at: https://www.mdpi.com/article/10.3390/vetsci11050206/s1, Figure S1: Vimentin WB images after up-/down-regulation of miR-329b-5p. Figure S2: PCNA WB images after up-/down-regulation of miR-329b-5p.
