# Supplementary material for: miR-329b-5p Affects Sheep Intestinal Epithelial Cells against Escherichia coli F17 Infection
Source: Vet Sci. 2024 May 8;11(5):206. doi: 10.3390/vetsci11050206 (PMC11126089; doi:10.3390/vetsci11050206)
Supplement: Supplementary file 1 [file vetsci-11-00206-s001.zip › vetsci-2976988-Supplementary Materials.pdf]

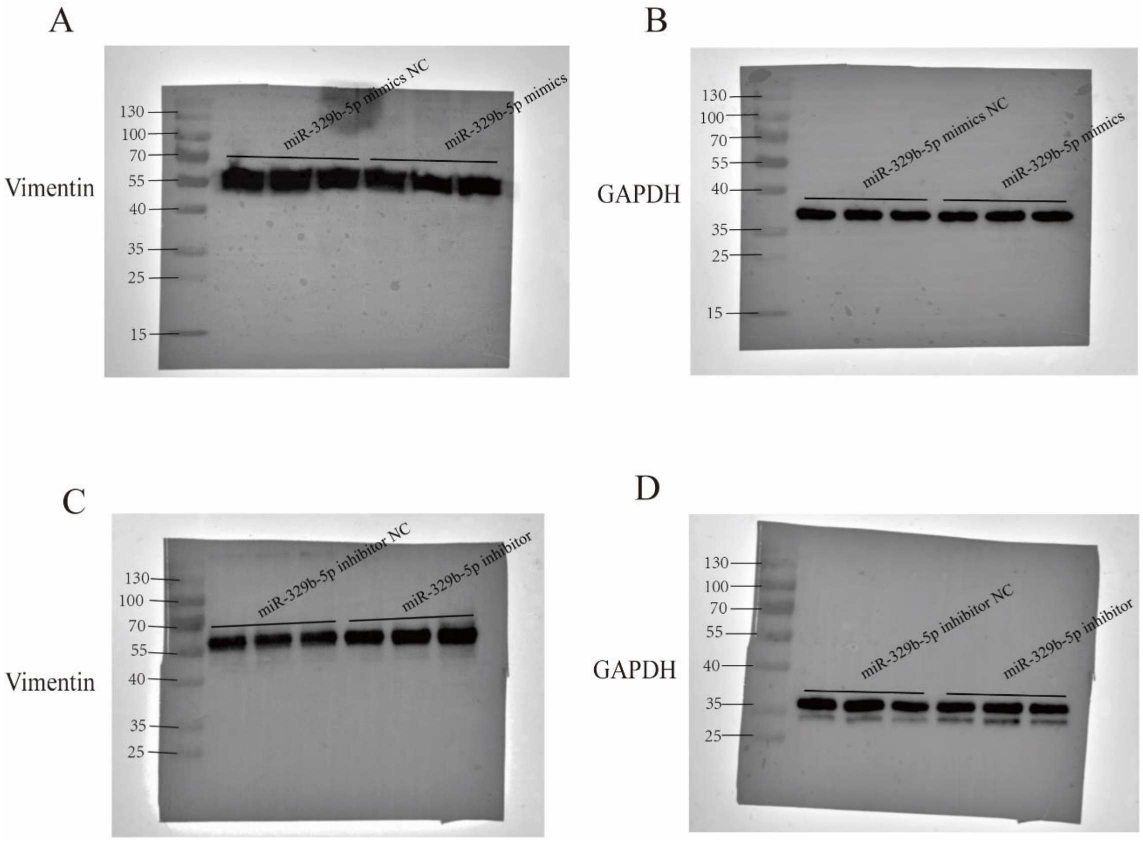

Figure S1. Vimentin WB images after up-/down-regulation of miR-329b-5p. (A) Vimentin WB image after up-regulation of miR-329b-5p. (B) Vimentin WB internal reference GAPDH image after up-regulation of miR-329b-5p. (C) Vimentin WB image after down-regulation of miR-329b-5p. (D) Vimentin WB internal reference GAPDH image after down-regulation of miR-329b-5p.

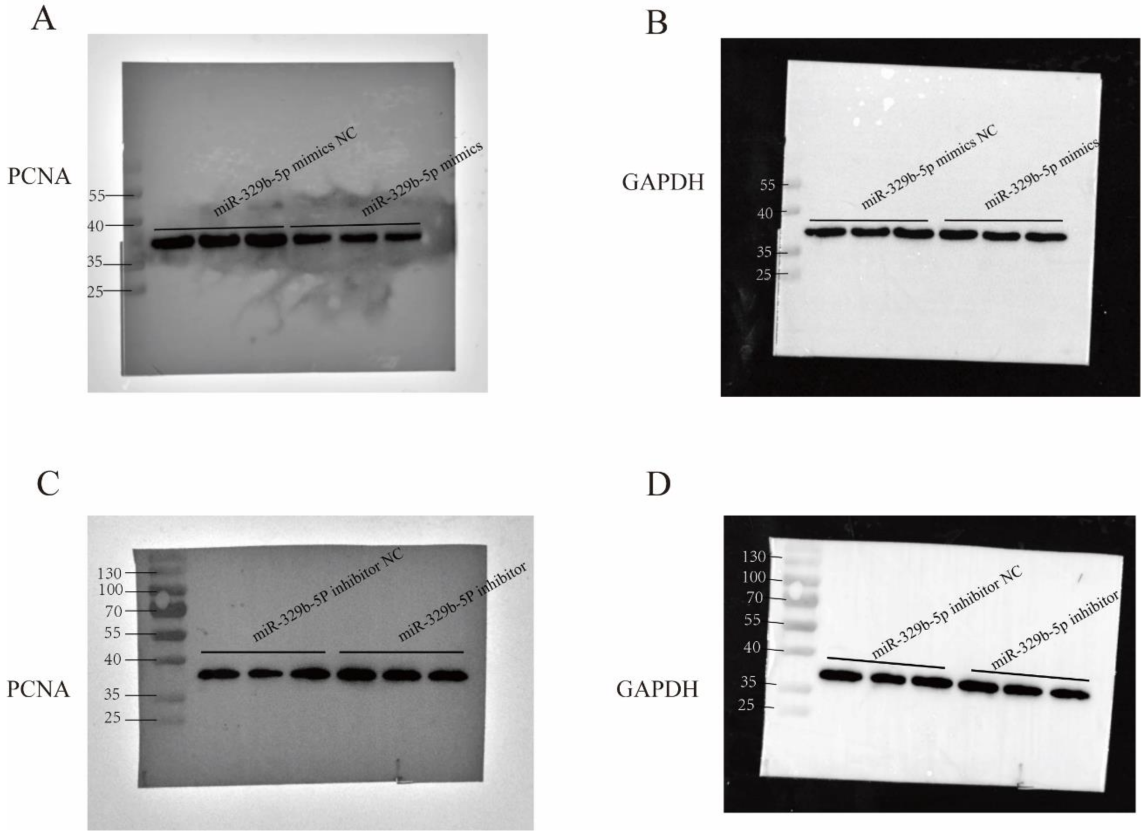

Figure S2. PCNA WB images after up-/down-regulation of miR-329b-5p. (A) PCNA WB image after up-regulation of miR-329b-5p. (B) PCNA WB internal reference GAPDH image after up-regulation of miR-329b-5p. (C) PCNA WB image after down-regulation of miR-329b-5p. (D) PCNA WB internal reference GAPDH image after down-regulation of miR-329b-5p.
